# A Human Antibody That Binds to the Sixth Ig-Like Domain of VCAM-1 Blocks Lung Cancer Cell Migration In Vitro

**DOI:** 10.3390/ijms18030566

**Published:** 2017-03-06

**Authors:** Mi Ra Kim, Ji Hye Jang, Chang Sik Park, Taek-Keun Kim, Youn-Jae Kim, Junho Chung, Hyunbo Shim, In Hyun Nam, Jung Min Han, Sukmook Lee

**Affiliations:** 1 Research Center, Scripps Korea Antibody Institute, Chuncheon 24341, Korea; cslove526@skai.or.kr (M.R.K.); jjh717@skai.or.kr (J.H.J.); pcs@skai.or.kr (C.S.P.); tkkim@skai.or.kr (T.-K.K.); 2 Specific Organs Cancer Branch, Research Institute, National Cancer Center, Goyang 10408, Korea; yjkim76@gmail.com; 3 Department of Biochemistry and Molecular Biology, Seoul National University, Seoul 03087, Korea; junhochung@icloud.com; 4 Departments of Bioinspired Science and Life Science, Ewha Womans University, Seoul 03760, Korea; hshim@ewha.ac.kr; 5 Geologic Environment Division, Korea Institute of Geoscience and Mineral Resources (KIGAM), Daejeon 34132, Korea; nih@kigam.re.kr; 6 Department of Integrated OMICS for Biomedical Science, College of Pharmacy, Yonsei University, Incheon 21983, Korea; jhan74@yonsei.ac.kr

**Keywords:** human antibody, invasion, lung cancer, Matrigel, migration, VCAM-1, VCAM-1-D6

## Abstract

Vascular cell adhesion molecule-1 (VCAM-1) is closely associated with tumor progression and metastasis. However, the relevance and role of VCAM-1 in lung cancer have not been clearly elucidated. In this study, we found that VCAM-1 was highly overexpressed in lung cancer tissue compared with that of normal lung tissue, and high VCAM-1 expression correlated with poor survival in lung cancer patients. VCAM-1 knockdown reduced migration of A549 human lung cancer cells into Matrigel, and competitive blocking experiments targeting the Ig-like domain 6 of VCAM-1 (VCAM-1-D6) demonstrated that the VCAM-1-D6 domain was critical for VCAM-1 mediated A549 cell migration into Matrigel. Next, we developed a human monoclonal antibody specific to human and mouse VCAM-1-D6 (VCAM-1-D6 huMab), which was isolated from a human synthetic antibody library using phage display technology. Finally, we showed that VCAM-1-D6 huMab had a nanomolar affinity for VCAM-1-D6 and that it potently suppressed the migration of A549 and NCI-H1299 lung cancer cell lines into Matrigel. Taken together, these results suggest that VCAM-1-D6 is a key domain for regulating VCAM-1-mediated lung cancer invasion and that our newly developed VCAM-1-D6 huMab will be a useful tool for inhibiting VCAM-1-expressing lung cancer cell invasion.

## 1. Introduction

Lung cancer is one of the more common types of cancer, and it is the leading cause of cancer death among men and the second leading cause of cancer death among women worldwide [1]. Conventionally, patients with lung cancer are treated by surgical resection, platinum-based chemotherapy, and radiation therapy, alone or in combination [2]. Recently, epidermal growth factor receptor (EGFR) and anaplastic lymphoma kinase (ALK) have been determined to be the most effective molecules for targeted therapy in lung cancer. *EGFR* mutations and *ALK* gene rearrangements are successfully targeted with specific tyrosine kinase inhibitors, including erlotinib, gefitinib, and crizotinib [3,4]. Furthermore, bevacizumab, a humanized anti-vascular endothelial growth factor (VEGF) antibody, is being used in clinics to treat lung cancer [5]. However, despite the current availability of therapeutic regimens, the major obstacle to overcome in lung cancer treatment is lung cancer cell invasion and metastasis. During these processes, malignant tumors cells first intravasate into blood vessels and then extravasate into new tissues, where they can proliferate and produce a metastatic secondary tumor [6,7]. Therefore, the identification of novel targets in lung cancer cell invasion is critical for developing more effective therapeutic options for treating lung cancer patients.

Vascular cell adhesion molecule-1 (VCAM-1) is a 90-kDa glycoprotein that is predominantly expressed on activated endothelial cells in response to pro-inflammatory cytokines, including human tumor necrosis factor alpha (hTNFα) [8,9]. VCAM-1 is a type I transmembrane protein that consists of an extracellular domain, with seven homologous immunoglobulin (Ig)-like domains, a transmembrane domain, and a cytosolic domain [10]. During an inflammatory response, α4β1 integrin-expressing leukocytes adhere to VCAM-1-expressing endothelial cells, which promote their transmigration across the activated endothelium [11]. Previously, we showed that the sixth Ig-like domain of VCAM-1 (VCAM-1-D6) is important for mediating this leukocyte transmigration, implying that it may play a role in VCAM-1-mediated inflammation [12].

Recently, some groups have also suggested roles for VCAM-1 in tumor progression and metastasis. VCAM-1 is overexpressed in several types of cancers, including renal, gastric, pancreatic, breast, and ovarian cancers [13,14,15,16,17,18]. Moreover, VCAM-1 expression in breast cancer cells enhances their metastasis to the lungs by allowing them to interact with leukocytes that express α4 integrin counter-receptors [15]. VCAM-1 expression is also associated with oncogenesis, tumor angiogenesis, and metastasis in gastric carcinoma [16]. However, the role of VCAM-1 and its target domain for antibody therapy in lung cancer cell invasion have not yet been clearly identified.

In the present study, we showed that VCAM-1 expression is increased in lung cancer tissue compared with that of normal lung tissue, and that high VCAM-1 expression is associated with reduced survival of lung cancer patients. Moreover, siRNA-mediated VCAM-1 knockdown and competitive inhibition experiment using recombinant VCAM-1-D6 protein demonstrated that VCAM-1 is required for lung cancer cell migration into Matrigel and that the VCAM-1-D6 domain of VCAM-1 is a key domain for regulating lung cancer cell migration into Matrigel. Finally, by developing of a VCAM-1 blocking monoclonal antibody specific to VCAM-1-D6, we found that the antibody specifically inhibited the lung cancer cell migration into Matrigel. In summary, this study provides proof-of-concept evidences showing a role for VCAM-1-D6 as a key domain in lung cancer cell invasion. Furthermore, the antibody-based targeting of VCAM-1-D6 is an effective strategy for inhibiting the invasion of VCAM-1-expressing lung cancer cells.

## 2. Results

### 2.1. VCAM-1 Expression Is Increased in Lung Cancer and Is Associated with Reduced Survival 

**Figure 1:**
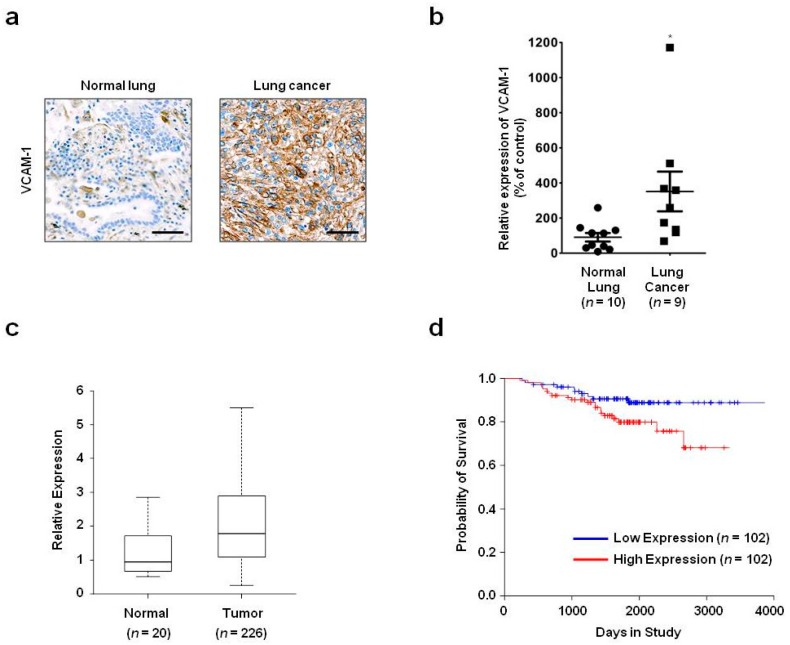
Expression and survival analyses of vascular cell adhesion molecule-1 (VCAM-1) in lung cancer patient samples. (**a**) Representative images are shown of the immnohistochemical examination of VCAM-1 in normal lung (*n* = 10) and lung cancer (*n* = 9) tissues using a commercially available anti-VCAM-1 antibody (scale bar = 200 µm); (**b**) A dot plot shows quantification of the immunohistochemical analyses of VCAM-1. Each dot represents the average of VCAM-1 expression levels quantified from three independent areas/sample. The values represent the mean ± SEM of the VCAM-1 expression calculated from each sample (* *p* < 0.05); (**c**) A box plot illustrates the results from VCAM-1 expression analyses that was performed with lung cancer patient gene expression profiling data obtained from the NCBI Gene Expression Omnibus database; (**d**) A Kaplan–Meier plot shows the overall survival of lung cancer patients classified by their VCAM-1 expression level (red: high expression group, *n* = 102; blue: low expression group, *n* = 102).

To investigate VCAM-1 expression in normal lung and lung cancer patient tissue, we performed immunohistochemistry with a commercially available anti-VCAM-1 antibody. VCAM-1 expression was higher in lung cancer (*n* = 9) compared with that in normal lung (*n* = 10) tissue (Figure 1a,b). To further examine VCAM-1 expression in normal lung and lung cancer patient tissue, we analyzed lung cancer patient gene expression profiling data (GSE31210) obtained from the National Cancer for Biotechnology Information (NCBI) Gene Expression Omnibus (GEO) database. VCAM-1 expression was significantly increased in lung cancer tissue (*n* = 226) compared with that in normal lung tissue (*n* = 20) (*p* = 0.00015, Figure 1c). Taken together, these expression data suggest that increased levels of VCAM-1 play a role in lung cancer.

To elucidate the relationship between VCAM-1 expression and survival of lung cancer patients, we performed bioinformatics-based survival analysis. The five-year overall survival rate was 91% in the low-VCAM-1-expression group (*n* = 102), whereas this rate was significantly reduced to 80% in the high-VCAM-1-expression group (*n* = 102) (*p* = 0.0493, Figure 1d). These data demonstrate an association between high VCAM-1 expression and poor survival and further suggest that increased VCAM-1 expression is closely associated with lung cancer.

### 2.2. The VCAM-1-D6 Domain of VCAM-1 Plays a Key Role in Regulating A549 Lung Cancer Cell Invasion

**Figure 2:**
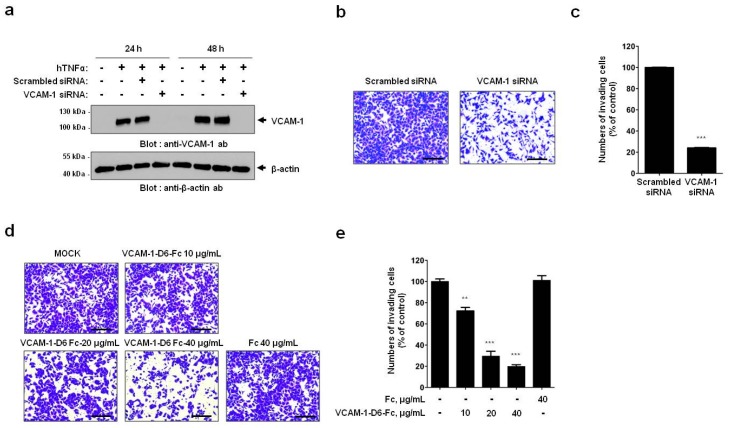
Modulation of VCAM-1 levels and function affect A549 lung cancer cell migration into Matrigel. (**a**) Representative immunoblots show the expression of VCAM-1 from scrambled siRNA- or VCAM-1 siRNA-transfected A549 cells cultured in the presence of hTNFα; (**b**) Representative images depict the migration of the scrambled siRNA- or VCAM-1 siRNA-transfected A549 cells into Matrigel cultured in the presence of hTNFα (200× magnification, scale bar = 200 µm); (**c**) Migrating cells into Matrigel were quantified in the scrambled siRNA- or VCAM-1 siRNA-transfected A549 cell groups and expressed as a percentage of the control values. All values represent the mean ± SEM of triplicate measurements from one of three independent experiments (*** *p* < 0.001); (**d**) Representative images illustrate the migration of VCAM-1-D6-Fc- or Fc-treated A549 cells into Matrigel cultured in the presence of hTNFα (200× magnification, scale bar = 200 µm); (**e**) The numbers of migrating cells into Matrigel in the VCAM-1-D6-Fc- or Fc-treated A549 cell groups were counted and expressed as a percentage of the control values. All values represent the mean ± SEM of triplicate measurements from one of three independent experiments (** *p* < 0.01, *** *p* < 0.001).

To examine the role of VCAM-1 in lung cancer cell invasion, we performed siRNA-mediated knockdown of VCAM-1 in the human lung cancer cell line A549, which was cultured in the presence of hTNFα to induce VCAM-1. First, we confirmed the reduced levels of VCAM-1 in VCAM-1 siRNA-, compared with scrambled siRNA-, transfected cells using immunoblot analysis (Figure 2a). Importantly, VCAM-1 knockdown significantly decreased A549 cell migration into Matrigel compared with that of control knockdown cells (*p* < 0.001, Figure 2b,c). Collectively, these results suggest that VCAM-1 regulates lung cancer cell invasion. 

Previously, we reported that VCAM-1-D6 is important for the transendothelial cell migration of leukocytes [12]. To investigate whether the VCAM-1-D6 domain plays a similar key role in the invasion of lung cancer cells, we prepared an Fc fusion protein of VCAM-1-D6 (VCAM-1-D6-Fc) and performed a competitive blocking experiment by treating A549 cells, grown in the presence of hTNFα with VCAM-1-D6-Fc, or Fc alone as a negative control. Importantly, A549 cell migration into Matrigel was inhibited in a dose-dependent manner by the addition of VCAM-1-D6-Fc, but not by Fc, compared with that of mock-treated cells (Figure 2d,e). To ensure that these effects on cell invasion are not due to effects on cell viability, we confirmed that VCAM-1 siRNA and VCAM-1-D6-Fc treatment did not affect A549 cell viability relative to their corresponding controls, whereas 5-fluorouracil (5-FU), an apoptosis inducer, significantly reduced A549 cell viability (Figure S1). These results suggest that the VCAM-1-D6 domain is crucial for VCAM-1-mediated lung cancer cell invasion. 

### 2.3. Development and Characterization of a Human Monoclonal Antibody Specific to VCAM-1-D6 of Human and Mouse VCAM-1 

To isolate human monoclonal antibodies to human and mouse VCAM-1-D6, a human synthetic single-chain variable fragment (scFv) library was pre-cleared to remove Fc binders. Phage enzyme-linked immunosorbent assays (ELISA) confirmed that approximately 90% of the scFvs binding to Fc were removed (data not shown). The library was alternately biopanned with human (hVCAM-1-D6-Fc) or mouse (mVCAM-1-D6-Fc) VCAM-1-D6 fusion proteins using VCAM-1-D6-Fc-coated magnetic beads. 96 phage clones were randomly selected, rescued by helper phage infection, and tested for reactivity to human and mouse VCAM-1-D6 using phage enzyme immunoassays. Following DNA sequencing, two clones, recognizing both human and mouse VCAM-1-D6, with different complementarity determining region sequences, were selected and converted to IgGs. They were expressed in human embryonic kidney (HEK) 293F cells and purified. SDS-PAGE and Coomassie staining showed that all IgG clones were greater than 90% pure (data not shown).

**Figure 3:**
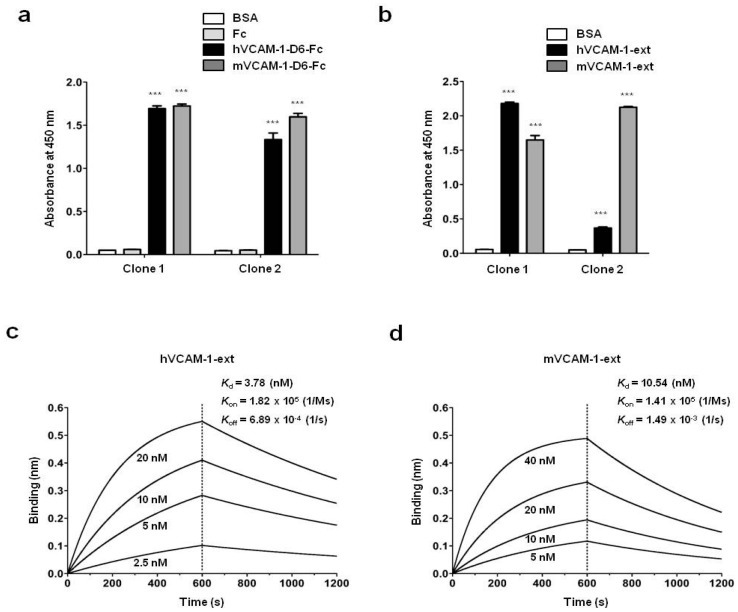
Generation and characterization of VCAM-1-D6 huMabs specific to human and mouse VCAM-1-D6. Bar graphs illustrate the binding specificity of VCAM-1-D6 huMab Clones 1 and 2 to hVCAM-1-D6-Fc, mVCAM-1-D6-Fc, and Fc (**a**), or to hVCAM-1-ext and mVCAM-1-ext (**b**). Following blocking with BSA, the binding specificities of VCAM-1-D6 huMabs to each of these proteins, which were coated onto 96-well microtiter plates, were measured by ELISA. The values represent the mean ± SEM of triplicate measurements from three independent experiments (*** *p* < 0.001). The binding affinities of VCAM-1-D6 huMab (Clone 1) to hVCAM-1-ext (**c**) and mVCAM-1-ext (**d**) were measured using a biolayer interferometry assay with the Octet^®^ RED96 system.

To investigate the cross-species reactivity of purified human monoclonal antibodies specific to VCAM-1-D6 (VCAM-1-D6 huMabs), we coated 96-well microtiter plates with hVCAM-1-D6-Fc, mVCAM-1-D6-Fc, or Fc and performed ELISAs. The VCAM-1-D6 huMabs specifically bound to human and mouse VCAM-1-D6-Fc, but not to Fc alone (Figure 3a). To further confirm their binding specificity, we coated 96-well microtiter plates with purified human and mouse VCAM-1 extracellular domains (hVCAM-1-ext and mVCAM-1-ext) and performed ELISAs. Clone 1 showed strong binding to hVCAM-1-ext and mVCAM-1-ext, whereas Clone 2 showed a weaker binding affinity to hVCAM-1-ext than to mVCAM-1-ext (Figure 3b). Thus, for the remainder of the study, Clone 1 was used as a representative of human monoclonal antibodies to VCAM-1-D6 (hereafter referred to as VCAM-1-D6 huMab). Finally, to more precisely measure the binding affinity of VCAM-1-D6 huMab to human and mouse VCAM-1, we performed a label-free kinetic analysis using the Octet biolayer interferometry system. The Kds of VCAM-1-D6 huMab (Clone 1) binding to hVCAM-1-ext and mVCAM-1-ext were approximately 3.78 and 10.54 nM, respectively (Figure 3c,d).

### 2.4. VCAM-1-D6 huMab Specifically Inhibits the Invasion of VCAM-1-Expressing Lung Cancer Cells

**Figure 4:**
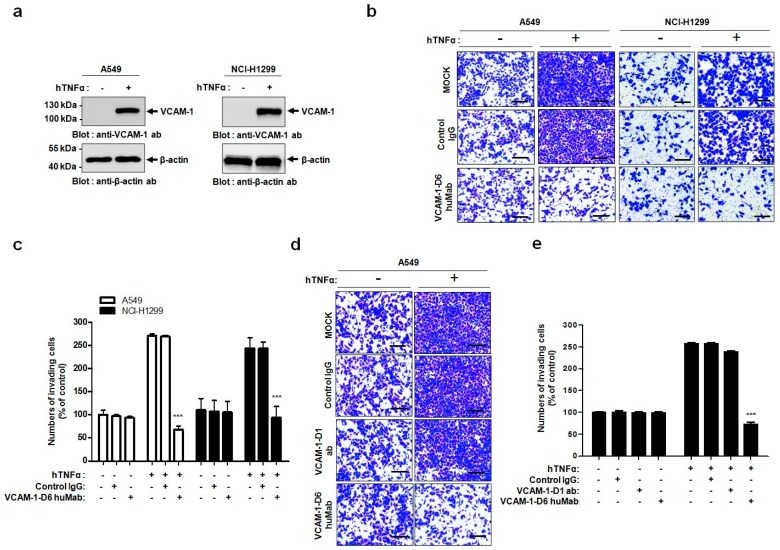
Effects of VCAM-1-D6 huMab on lung cancer cell migration into Matrigel. (**a**) Representative immunoblots show the expression of VCAM-1 in A549 and NCI-H1299 lung cancer cells in the absence or presence of hTNFα; (**b**) Representative images highlight the effects of VCAM-1-D6 huMab treatment on A549 and NCI-H1299 cell migration into Matrigel, in the absence or presence of hTNFα, compared with those of control IgG treatment (200× magnification, scale bar = 200 µm); (**c**) The numbers of A549 and NCI-H1299 migrating cells into Matrigel in the presence of control IgG or VCAM-1-D6 huMab are expressed as a percentage of the mock control values. The values represent the mean ± SEM of triplicate measurements from one of three independent experiments (*** *p* < 0.001); (**d**) Representative images highlight the specific effects of VCAM-1-D6 huMab on A549 cell migration into Matrigel, in the absence or presence of hTNFα, compared with those of a VCAM-1-D1 blocking antibody or control IgG (200× magnification, scale bar = 200 µm); (**e**) The numbers of migrating cells into Matrigel in the presence of control IgG, VCAM-1-D1 blocking antibody, and VCAM-1-D6 huMab are expressed as a percentage of the mock control values. The values represent the mean ± SEM of triplicate measurements from one of two independent experiments (*** *p* < 0.001).

Prior to performing functional assays, we verified VCAM-1 expression in the human lung cancer cell lines A549 and NCI-H1299 that were cultured in the absence or presence of hTNFα. Immunoblot analysis showed that VCAM-1 expression was dramatically increased in the A549 and NCI-H1299 cells in response to hTNFα (Figure 4a). Next, to investigate the effect of VCAM-1-D6 huMab on lung cancer cell invasion, we treated these cells cultured in the absence or presence of hTNFα with VCAM-1-D6 huMab, or IgG as a negative control, and then, we performed invasion assays. The migration of A549 and NCI-H1299 cells into Matrigel (Figure 4b,c) was specifically inhibited by VCAM-1-D6 huMab, but not by the IgG control antibody. 

To further confirm the specific inhibitory effect of VCAM-1-D6 huMab on lung cancer cell invasion, we treated A549 cells, cultured in the absence or presence of hTNFα, with VCAM-1-D6 huMab or a commercially available VCAM-1-D1 blocking antibody, and then we performed invasion assays. A549 cell migration into Matrigel was more potently inhibited by the VCAM-1-D6 huMab than by the VCAM-1-D1 blocking antibody (Figure 4d,e). These data further demonstrated the specific role of the VCAM-1-D6 domain in regulating VCAM-1-mediated lung cancer cell invasion, and suggest that the VCAM-1-D6 huMab can be used to suppress the invasion of VCAM-1-expressing lung cancer cells in vivo.

## 3. Discussion

Lung cancer is one of the leading causes of cancer death worldwide [1]. Despite the remarkable advances in lung cancer therapeutics, lung cancer cell invasion and metastasis remain the major obstacles to overcome to improve patient survival. Thus, increasing attention is focused on identifying novel molecules that are differentially expressed and/or play a key role in lung cancer cell invasion. Although the roles of VCAM-1 have been reported in several types of cancer [13,14,15,16,17,18], the roles of VCAM-1 in lung cancer have not yet been elucidated. More specifically, the role of the VCAM-1-D6 domain in lung cancer invasion has not been explored.

In the present study, we propose, for the first time, that the VCAM-1-D6 domain may be a key domain for regulating VCAM-1-mediated lung cancer cell invasion. Several lines of evidence support our proposal. First, compared with normal lungs, VCAM-1 was highly overexpressed at the mRNA and protein level in lung cancer tissue. Moreover, although further studies are needed to verify all the cell types that express VCAM-1 in lung cancer tissue, the uniformly strong expression of VCAM-1, as detected by immunohistochemical staining, throughout the lung cancer tissue was quite distinct from the highly specific endothelial staining pattern in lung cancer tissue (Figure S2). This result suggests that most VCAM-1-stained cells are cancer cells, rather than endothelium. However, we cannot exclude the possibility that other cell types also express VCAM-1 in lung cancer tissue. Second, high VCAM-1 expression correlated with poor survival of lung cancer patients, implying that VCAM-1 has a key role in lung cancer progression. Third, siRNA-mediated knockdown of VCAM-1 in hTNFα-treated A549 human lung cancer cells greatly reduced cell migration into Matrigel, suggesting VCAM-1 is critical for this process. Fourth, using competitive inhibition assays with recombinant VCAM-1-D6, we identified VCAM-1-D6 as a key domain regulating A549 cell migration into Matrigel. Fifth, by developing a VCAM-1-D6 blocking antibody, VCAM-1-D6 huMab, we demonstrated that antibody-based inhibition of VCAM-1-D6 effectively reduced VCAM-1-mediated migration of A549 and NCI-H1299 lung cancer cell lines into Matrigel. Sixth, we confirmed that A549 cell migration into Matrigel was more potently inhibited by the VCAM-1-D6 huMab than by a blocking antibody to the VCAM-1-D1 domain (51-10C9), demonstrating the specificity of the role of VCAM-1-D6 in lung cancer cell migration into Matrigel. Finally, we also confirmed that VCAM-1-D6 huMab and VCAM-1-D6 chimeric monoclonal antibody (VCAM-1-D6 chimeric Mab), which we previously developed [12], have similar inhibitory effects on A549 cell migration into Matrigel and on U937 transendothelial migration across activated human umbilical vein endothelial cells (HUVECs) (Figure S3). These findings further support the importance, and the efficiency and specificity, of antibody-based modulation of VCAM-1-D6 on lung cancer cell invasion. Our study focused on elucidating the role of the VCAM-1-D6 domain in VCAM-1-mediated lung cancer cell invasion using domain-specific recombinant protein and antibodies; however, we cannot exclude the possibility that other domains of VCAM-1 and other associated regulatory molecules including metalloproteinases also contribute to this process.

In this study, we generated a human antibody to VCAM-1-D6, which is the functionally critical domain for VCAM-1-mediated lung cancer cell migration into Matrigel. Conventional antibody screening uses the whole extracellular domain of a protein as a target antigen; thus, most selected antibodies bind to the target protein but are not necessarily functional. Therefore, the identification of functional domains would drastically facilitate the time-consuming and labor-intensive functional antibody screening process. Here, we demonstrated a more effective strategy to develop a functional antibody by first identifying VCAM-1-D6 as a key domain regulating VCAM-1-mediated lung cancer cell migration into Matrigel and subsequently isolating a VCAM-1-D6-specific antibody from a human synthetic antibody library. The antibody isolated using this strategy, VCAM-1-D6 huMab, efficiently inhibited VCAM-1-mediated lung cancer cell migration into Matrigel. Moreover, VCAM-1-D6 huMab is a human antibody, which makes it is likely to be less immunogenic in vivo, and it has a nanomolar affinity. Both of these qualities may make VCAM-1-D6 huMab an attractive candidate therapeutic for potential use in combination with existing chemotherapies to suppress the invasion of VCAM-1-expressing lung cancer cells in human lung cancer patients. 

VCAM-1 regulates many distinct cell processes by specifically interacting with various proteins, including α4β1 integrin, CD44, moesin, ezrin, and secreted protein acidic and rich in cysteine (SPARC), depending on the spatiotemporal environment of a particular cell [19,20,21,22]. Here, we showed that lung cancer cell migration into Matrigel was specifically and prominently inhibited by VCAM-1-D6 huMab but not by a VCAM-1-D1 blocking antibody. These data strongly suggest that the VCAM-1-D1 domain, which interacts with α4β1 integrin expressed on leukocytes to regulate leukocyte recruitment during the initiation and progression of inflammation [8,20], does not play a role in VCAM-1-mediated lung cancer cell invasion. It is well known that antibody-induced internalization often leads to the downregulation of target proteins. Moreover, we observed that VCAM-1-D6 huMab treatment did not alter the level of VCAM-1 expressed on the surface of VCAM-1 expressing cells (Figure S4). These combined results lead us to speculate that VCAM-1-D6 huMab likely acts as a VCAM-1-D6-blocking antibody by binding to VCAM-1-D6, potentially altering its structure, and resulting in inhibition of VCAM-1-mediated signaling that is necessary for lung cancer cell invasion; however, further studies are required to address the precise binding and signaling effects of VCAM-1-D6 huMab. In conclusion, VCAM-1-D6 huMab may be used as a valuable research tool for identifying the role of VCAM-1 via the VCAM-1-D6 domain, and for elucidating the detailed molecular mechanisms of VCAM-1-mediated lung cancer cell invasion. 

**Figure 5:**
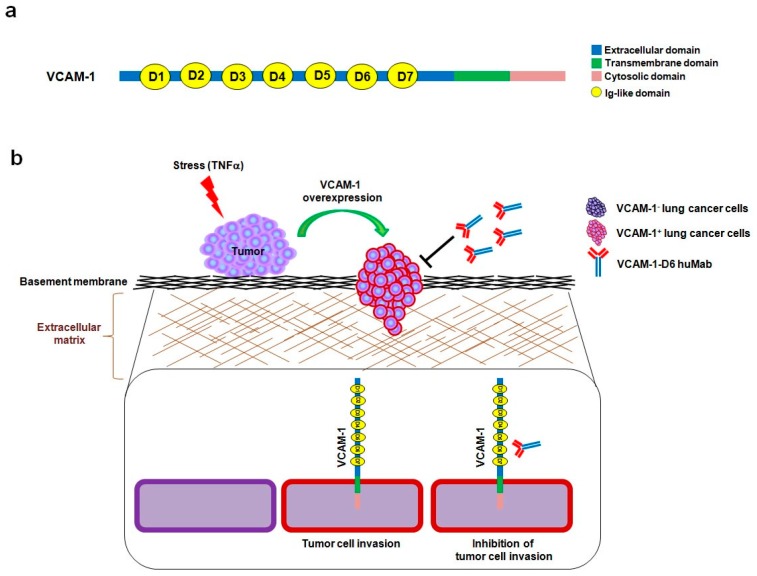
Schematic representation of the inhibitory mechanism of VCAM-1-D6 huMab in lung cancer cell invasion. (**a**) A schematic diagram illustrates the primary structure of VCAM-1, which is comprised of an extracellular domain with seven Ig-like domains (D1~D7), a transmembrane domain, and a cytosolic domain; (**b**) A proposed model of the inhibitory mechanism of VCAM-1-D6 huMab on lung cancer cell invasion is shown. Under stressful conditions, such as the abrupt elevation of TNFα levels within the tumor microenvironment, VCAM-1 levels are dramatically increased on the surface of lung cancer cells. The VCAM-1-D6 domain of VCAM-1 plays a key role in lung cancer cell invasion; however, the addition of VCAM-1-D6 huMab specifically inhibits VCAM-1-mediated lung cancer cell invasion.

In summary, this study provides proof-of-concept evidence of the utility of developing a domain-specific antibody, following the identification of a key functional protein domain, for antibody-based targeting in cancer therapy. Here, we identified VCAM-1-D6 as a key domain for regulating lung cancer cell migration into Matrigel, and we demonstrated the efficacy of domain-specific, antibody-based targeting of VCAM-1-D6 to inhibit the migration of VCAM-1-expressing human lung cancer cells into Matrigel. Based on our data, we propose a mode of action whereby, under stressful conditions within the tumor microenvironment and in response to certain extracellular stimuli, VCAM-1 expression is abruptly increased on the surface of lung cancer cells to promote lung cancer cell invasion. VCAM-1-D6 huMab binds to VCAM-1 and blocks the invasion of VCAM-1-expressing human lung cancer cells (Figure 5). In future studies, we will investigate the precise mechanism of the VCAM-1-D6 domain in VCAM-1-mediated cell invasion and evaluate the in vivo efficacy of VCAM-1-D6 huMab in lung cancer animal models.

## 4. Materials and Methods 

### 4.1. Survival Analysis 

Lung cancer patient gene expression profiling data (GSE31210) were obtained from the National Center for Biotechnology Information (NCBI) Gene Expression Omnibus (GEO) database. The lung cancer patients were classified into two groups, low- or high-VCAM-1-expression, based on whether their median VCAM-1 expression level was lower than or higher than the median VCAM-1 expression level of all patients, respectively. The Kaplan–Meier analysis and log-rank test were performed using the R survival package.

### 4.2. Immunohistochemistry 

Immunohistochemistry was performed as described previously with minor modifications [23]. Briefly, tissue slides printed with normal lung or lung cancer tissues were purchased from SuperBioChips Laboratories (Seoul, Korea). The slides were incubated first with mouse anti-VCAM-1 monoclonal antibody (1:200; Abcam, Cambridge, MA, USA) and then with biotinylated goat anti-mouse IgG (1:200; Vector Laboratories, Burlingame, CA, USA). Immunoreactive proteins were visualized using VECTASTAIN ABC Reagent (Vector Laboratories). For chromogenic reactions, the slides were incubated with fresh 3,3′-diaminobenzidine tetrahydrochloride solution (Vector Laboratories). All samples were counterstained with Meyer’s hematoxylin (Vector Laboratories). VCAM-1 expression was observed by light microscopy using an Olympus BX51 microscope (Olympus, Tokyo, Japan), and RGB images were acquired using Paint Shop Pro X software (Corel, Ottawa, ON, Canada). After performing background subtraction, VCAM-1 expression was quantified by measuring the signal density with Image J software version 1.48v (National Institutes of Health, Bethesda, MA, USA).

### 4.3. Cell Culture 

All lung cancer cell lines, including A549 and NCI-H1299 cells (Korean Cell Line Bank, Seoul, Korea) were cultured in RPMI1640 medium (Gibco, Waltham, MA, USA) supplemented with 10% (*v*/*v*) fetal bovine serum (FBS; Gibco) and 1% (*v*/*v*) penicillin/streptomycin (Gibco) according to the provider’s recommendations. Human umbilical vein endothelial cells (HUVECs; Lonza, Basel, Switzerland) were maintained in endothelial growth medium-2 (EGM-2; Lonza). All cells were maintained at 37 °C in a humidified incubator with 5% CO_2_ (Panasonic Healthcare Company, Tokyo, Japan). Throughout this study, all lung cancer cell lines and HUVECs were passaged less than five times and were used within 2 weeks of thawing. Expi293F™ cells (Gibco) were cultured in Expi293™ expression medium (Gibco) in a humidified Multitron incubator shaker (Infors HT, Basel, Switzerland) at 37 °C with 8% CO_2_. 

### 4.4. Transfection 

A549 cells were grown to 50%–80% confluence and transiently transfected with ON-TARGETplusSMARTpoolsiRNA targeting human VCAM-1 (GE Healthcare Dharmacon, Lafayette, CO, USA) using LipofectamineRNAiMAX transfection reagent (Invitrogen, Waltham, MA, USA) according to the manufacturer’s instructions.

### 4.5. Immunoblot Analysis 

Immunoblot analysis was performed as described previously with minor modifications [24]. Briefly, cell lysates were resolved by sodium dodecyl sulfate-polyacrylamide gel electrophoresis (SDS-PAGE) and transferred onto nitrocellulose membranes using a wet transfer system (GE Healthcare Life Sciences, Piscataway, NJ, USA). After blocking with Tris-buffered saline and Tween (TBST; 10 mM Tris-HCl, pH 7.5, 150 mM NaCl, and 0.05% (*v*/*v*) Tween 20) containing 5% (*w*/*v*) skim milk, the membranes were incubated with rabbit anti-VCAM-1 monoclonal antibody (1:1000; Abcam) or mouse anti-β-actin monoclonal antibody (1:1000; Santa Cruz Biotechnology, Santa Cruz, CA, USA) at 4 °C overnight, and then with horseradish peroxidase (HRP)-conjugated goat anti-rabbit IgG (1:5000; Cell Signaling Technology, Danvers, MA, USA) or horse anti-mouse IgG (1:5000; Cell Signaling Technology). Following several washes with TBST, protein bands were visualized using SuperSignal West Pico Chemiluminescent Substrate (Pierce, Rockford, IL, USA) according to the manufacturer’s instructions.

### 4.6. Construction and Preparation of Fc Fusion Proteins 

The DNA sequences encoding human VCAM-1-D6 (amino acids 511–595) and mouse VCAM-1-D6 (amino acids 511–595) were amplified by polymerase chain reaction (PCR). The PCR products were subcloned using two asymmetric SfiI sites into a modified pCEP4 mammalian expression vector (Invitrogen, Carlsbad, CA, USA) that contains the hinge and CH2-CH3 domains of human IgG1 at the 3′ end of the cloning site. The constructs were verified by DNA sequencing. The Fc fusion protein constructs were transfected into Expi293F™ cells using the ExpiFectamine™ Transfection Kit (Gibco). After seven days, the culture supernatants were collected, and the fusion proteins were purified using affinity chromatography with Protein A-Sepharose beads (GenScript, Piscataway, NJ, USA). After quantifying the protein concentrations using a NanoDrop 2000 (Thermo Fisher Scientific, Waltham, MA, USA) and performing dialysis in phosphate-buffered saline (PBS), the sample purity was analyzed by SDS-PAGE and Coomassie brilliant blue staining. The final pooled fractions were aliquoted and stored at −80 °C.

### 4.7. Selection of Human Antibodies Specific to VCAM-1-D6 Using Phage Display Technology

Biopanning to select human antibodies specific to VCAM-1-D6 from a synthetic human scFv antibody library was performed as described previously [25]. Briefly, following pre-clearing of Fc binders, 1 × 10^7^ magnetic beads (Dynabeads M-270 epoxy; Invitrogen) were coated with 4 μg recombinant human or mouse VCAM-1-D6-Fc for six rounds of biopanning. After the final round, 96 individual phage clones that displayed scFv were randomly selected from colonies grown on output plates. They were tested for their reactivity to human or mouse VCAM-1-D6-Fc using a phage enzyme immunoassay. The final enzyme-linked immunosorbent assay (ELISA)-positive scFv clones were analyzed by DNA sequencing, and two unique scFv clones with different complementaritydeterminingregion sequences were identified.

### 4.8. Preparation of VCAM-1-D6 huMabs

The variable heavy chain (V_H_) and variable light chain (V_L_) genes of the selected scFv clones (clone 1 and 2) were amplified using PCR. The V_H_ genes were engineered with a 5′ *Eco*RI site and a 3′ *Apa*I site, and the V_L_ with a 5′ *Hind*III site and a 3′ *Bsi*WI site. PCR fragments were digested with the appropriate restriction enzymes (New England BioLabs, Beverly, MA, USA) and cloned into the bicistronic mammalian expression vector pcDNA3.1 (Invitrogen), which encodes the CH1/hinge/CH2/CH3 domains of human IgG1 in the V_H_ cloning site and the constant kappa chain in the V_L_ cloning site. The VCAM-1-D6 huMabs were expressed and purified as described previously [21].

### 4.9. Transwell Invasion Assays 

Cell invasion assays were performed as described previously with minor modifications [24]. Briefly, 1.5 × 10^5^ A549 or NCI-H1299 cells were plated in 6-well plates, allowed to attach overnight, and treated with 20 ng/mL hTNFα (R&D systems, Minneapolis, MN, USA) for 24 h at 37 °C. For all cell invasion assays, 5 × 10^4^ A549 or NCI-H1299 cells were loaded onto the upper part of the transwell insert pre-coated with 100 µL Matrigel (Corning, Tewksbury, MA, USA), and the lower chamber was filled with complete medium containing 10% (*v*/*v*) FBS. To investigate the effects of VCAM-1 knockdown on cell invasion, transwell invasion assays were performed using scrambled siRNA- and VCAM-1 siRNA-transfected A549 cells. To verify the effects of the Fc fusion proteins on cell invasion, transwell invasion assays were performed in the presence of 0, 10, 20, or 40 μg/mL VCAM-1-D6-Fc fusion protein, or 40 μg/mL Fc domain alone, for 24 h at 37 °C. To check the ability of the anti-VCAM-1-D6 huMab to inhibit cell invasion, transwell invasion assays were performed in the presence of 20 μg/mL antibody [control IgG, anti-VCAM-1-D6 huMab, or VCAM-1-D1 blocking antibody (51-10C9, BD Bioscience)] for 24 h at 37 °C. Following the removal of non-invading cells by wiping the upper surface of the membrane with a cotton swab, the membrane was fixed with 4% (*v*/*v*) paraformaldehyde and stained with 0.2% (*w*/*v*) crystal violet solution. The degree of cell invasion was quantified by counting the number of cells that had migrated through the membrane in three random fields (200× magnification) per filter. 

### 4.10. Enzyme Linked Immunosorbent Assay (ELISA) 

ELISA was performed as described previously with minor modifications [26]. Briefly, 0.1 μg recombinant hVCAM-1-D6-Fc, mVCAM-1-D6-Fc, hVCAM-1 extracellular domain (hVCAM-1-ext; Sino Biological, Beijing, China), mVCAM-1 extracellular domain (mVCAM-1-ext; Sino Biological), or Fc was coated onto 96-well microplates and incubated overnight at 37 °C. Following three washes with PBS containing 0.05% (*v*/*v*) Tween 20 (PBST) and blocking with 3% (*w*/*v*) bovine serum albumin (BSA) in PBST for 1 h at 37 °C, the plates were incubated individually with 0.3 μg anti-VCAM-1-D6 huMab (Clone 1) or anti-VCAM-1-D6 huMab (Clone 2) in blocking buffer for 2 h at 37 °C. The plates were washed three times with PBST and then incubated with HRP-conjugated goat anti-human Fab antibody (1:3000; Thermo Fisher Scientific) in blocking buffer for 1 h at 37 °C. After three washes with PBST, 3,3′,5,5′-tetramethylbenzidine substrate solution (TMB; BD Biosciences, San Jose, CA, USA) was added to each well. The reaction was stopped by the addition of an equal volume of 1N H_2_SO_4_. The optical density was measured at 450 nm using a VICTOR™ X4 spectrophotometer (PerkinElmer, Waltham, MA, USA).

### 4.11. Real-Time Measurement of Antibody-Antigen Interactions 

A biolayer interferometry (BLI) assay was performed using an Octet^®^ RED96 system (ForteBio/Pall Life Sciences, Menlo Park, CA, USA) as described previously with minor modifications [24]. Briefly, hVCAM-1-ext or mVCAM-1-ext was immobilized onto amine-reactive biosensors (ForteBio) by amine coupling according to the manufacturer’s instructions. VCAM-1-D6 huMab was serially diluted 2-fold in 1× kinetics buffer. All interaction analyses were conducted at 30 °C in 1× kinetics buffer with 1000 rpm agitation. Association and dissociation (*K*_d_) constants were determined by 1:1 binding-model fitting using the ForteBio Octet Data Analysis software version 7.1 (ForteBio).

### 4.12. Statistical Analysis 

All data were analyzed with GraphPad Prism 5.0 (GraphPad Software, La Jolla, CA, USA) using a two-tailed Student’s *t*-test for comparison between two groups and a one-way analysis of variance with Bonferroni’s correction for multiple comparisons. All data are presented as the mean ± the standard error of the mean (SEM). A *p*-value < 0.05 was considered statistically significant.

## Supplementary Materials

Supplementary materials can be found at www.mdpi.com/1422-0067/18/3/566/s1.

## Abbreviations

huMab	Human monoclonal antibody
Ig	Immunoglobulin
VCAM-1	Vascular cell adhesion molecule-1
VCAM-1-D6	Sixth Ig-like domain of VCAM-1
