# Supplementary material for: A Human Antibody That Binds to the Sixth Ig-Like Domain of VCAM-1 Blocks Lung Cancer Cell Migration In Vitro
Source: Int J Mol Sci. 2017 Mar 6;18(3):566. doi: 10.3390/ijms18030566 (PMC5372582; doi:10.3390/ijms18030566)
Supplement: Supplementary file 1 [file ijms-18-00566-s001.pdf]

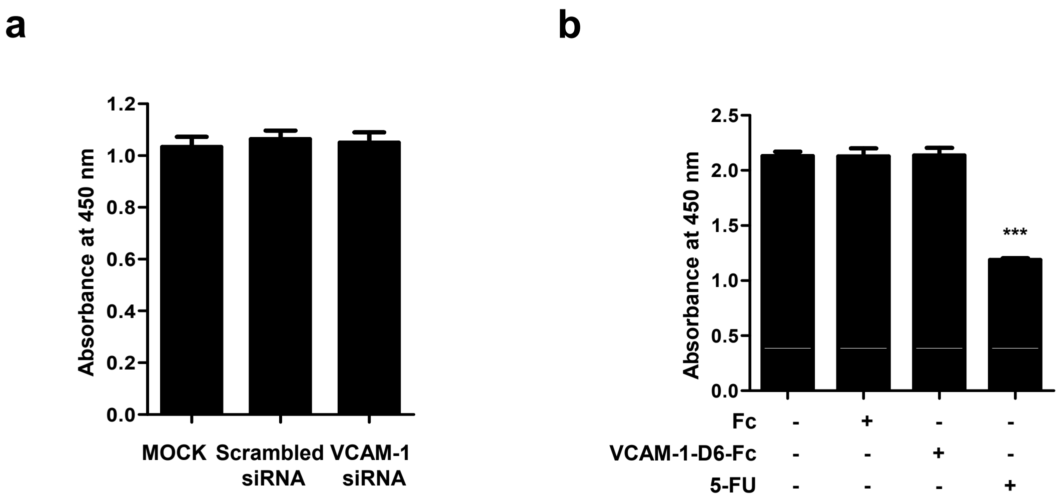

Figure S1. Kim et al.

Lung cancer

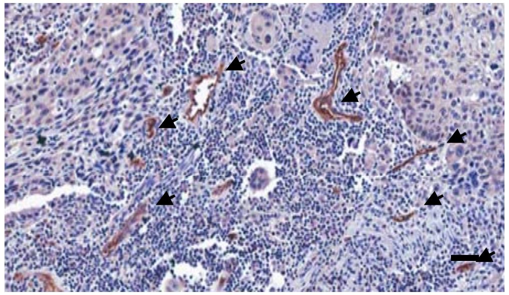

Figure S2. Kim et al.

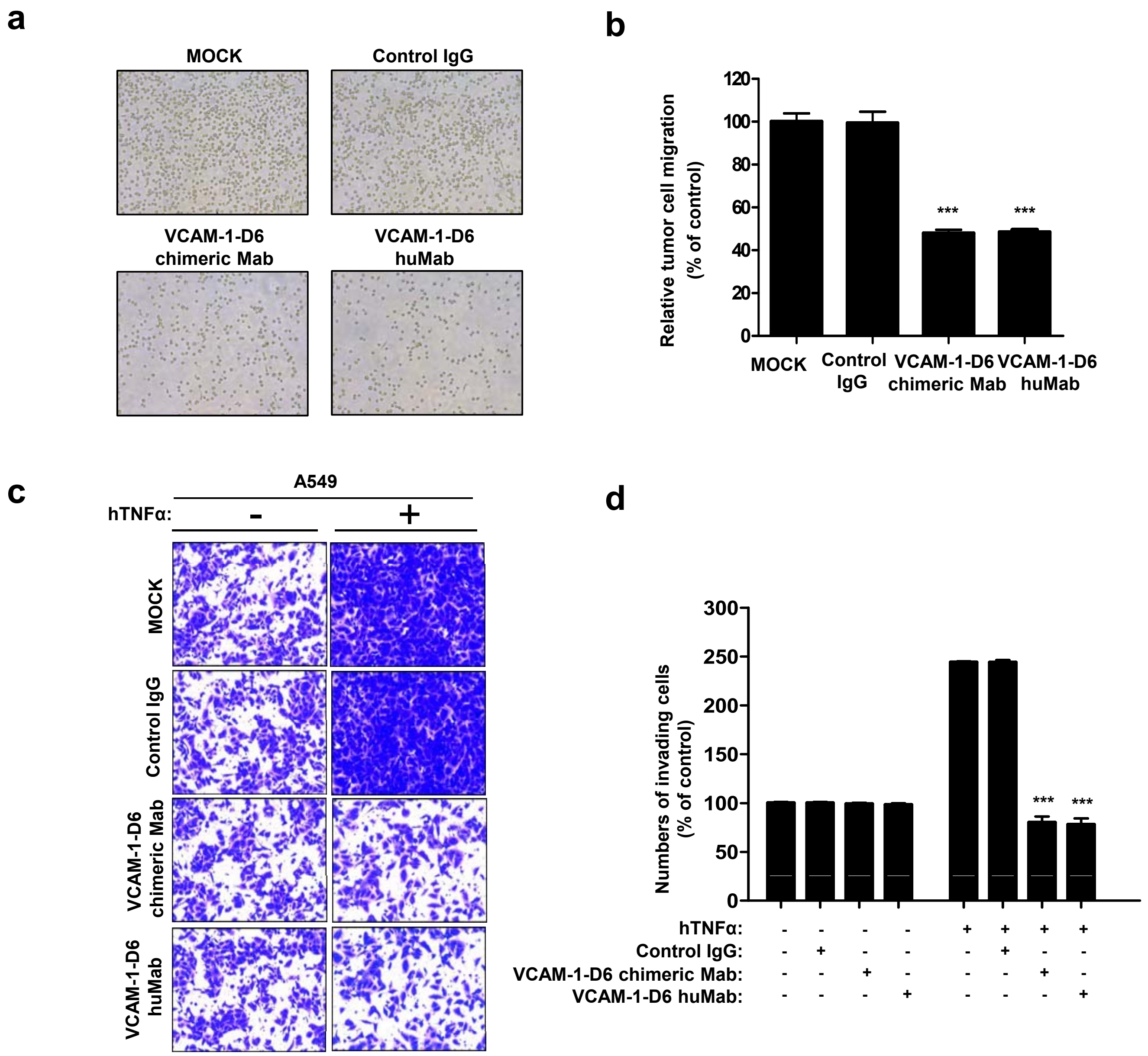

Figure S3. Kim et al.

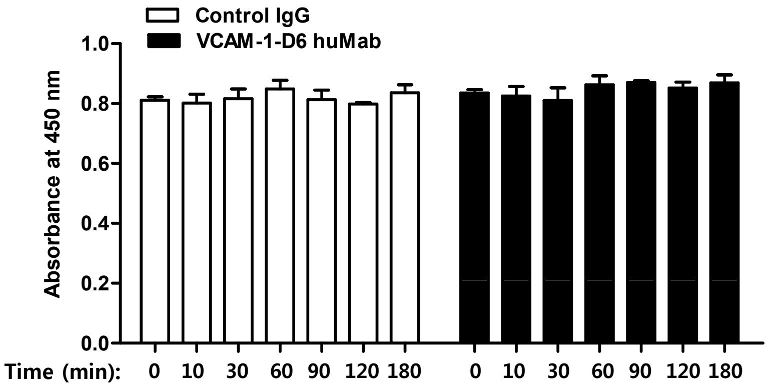

Figure S4. Kim et al.

Supplementary Figure Legends

Figure S1. Effects of VCAM-1 siRNA and VCAM-1-D6-Fc on TNFα-treated A549 cell viability. (a and b) After culturing A549 cells in the presence of 20 ng/ml hTNFα, the cells were incubated in the absence (MOCK) or presence of scramble siRNA or VCAM-1 siRNA for 1 d (a), or in the absence or presence of VCAM-1-D6-Fc (20 μg/ml), Fc (20 μg/ml), or 5-FU (36 μg/ml) for 1 d (b). Cell viability was assessed using the Cell Counting Kit-8 and measuring the absorbance at 450 nm. The data shown represent the mean ± SD from an experiment performed in triplicate (***$P<0.001$).

Figure S2. Immunohistochemical analysis of CLEC14a-positive tumor vessels in lung cancer patient tissue. Immunohistochemistry was performed with commercially available antibodies against CLEC14a, a tumor endothelial marker. The arrows show the CLEC14a-positive tumor vessels in lung cancer patient tissue counterstained with hematoxylin (scale bar=100 μm).

Figure S3. Comparative analysis of the effects of VCAM-1-D6 huMab and VCAM-1-D6 chimeric Mab on the transendothelial migration of U937 cells across TNFα-treated human umbilical vein endothelial cells (HUVECs) and on the migration of hTNFα-treated A549 cells into Matrigel. (a) Representative images depict the transendothelial migration of U937 cells across hTNFα-treated HUVECs in the presence or absence of VCAM-1-D6 huMab or VCAM-1-D6 chimeric Mab; (b) The numbers of migrated cells were quantified and expressed as a percentage of the control values; (c) Representative images depict the migration of the hTNFα-treated A549 cells into Matrigel in the presence or absence of

VCAM-1-D6 huMab or VCAM-1-D6 chimeric Mab; (d) The numbers of migrating cells into Matrigel were quantified and expressed as a percentage of the control values. All values represent the mean ± SD from an experiment performed in triplicate (***$P<0.001$).

Figure S4. Effect of VCAM-1-D6 huMab on VCAM-1 downregulation on the surface of VCAM-1-expressing cells. After culturing HUVECs in the presence of hTNFα, the cells were incubated in the presence of control IgG or VCAM-1-D6 huMab for the indicated times, and then, after several washings, VCAM-1 on the surface of the HUVECs was assayed by cell enzyme-linked immunosorbent assay (ELISA). The data shown represent the mean ± SD from an experiment performed in triplicate.

Supplementary Methods

1. Immunohistochemistry

Human lung tissue sections were obtained from SuperBiochips Laboratories (Korea). The tissue sections were incubated with sheep anti-CLEC14a antibody (1:500; R&D Systems). Then, the tissue sections were stained using the Anti-Sheep HRP-DAB Cell & Tissue Staining Kit (R&D Systems) and counterstained with hematoxylin (Vector Laboratories) according to the manufacturer's instructions. Images were obtained and processed using the Aperio Slide Scanner and ImageScope software (Leica Biosystems, San Diego, CA, USA).

2. Cell viability assay

Cell viability assays were performed as previously described (21). Briefly, before each experiment, A549 cells were treated with 20 ng/ml hTNFα for 1 d. To examine the effect of VCAM-1 knockdown on A549 cell viability, hTNFα-treated A549 cells (5×10^3) seeded in 96-well plates were incubated in the presence or absence of 20 nM scramble siRNA or VCAM-1 siRNA for 1 d. To investigate the effect of VCAM-1-D6-Fc on A549 cell viability, hTNFα-treated A549 cells (5×10^3) seeded in 96-well plates were incubated in the presence or absence of 20 μg/ml VCAM-1-D6-Fc, Fc, or 36 μg/ml 5-FU at 37°C for 1 d. Then, cell viability was measured using the Cell Counting Kit-8 (Dojindo, Kumamoto, Japan) according to manufacturer's instructions. Absorbance was measured at 450 nm with a VICTOR X4 spectrophotometer (Perkin Elmer, Waltham, MA, USA).

3. Transendothelial cell migration assay

Transendothelial cell migration assays were performed as previously described (12). Briefly, HUVECs (5×10^4) were added to the upper chambers of a 24-transwell plate

contained polycarbonate membranes with 8.0-mm diameter pores (Corning, Corning, NY, USA) and incubated overnight. Following the treatment of the cells with 20 ng/ml hTNFα for 1 d, 20 μg/ml VCAM-1-D6 huMab and VCAM-1-D6 chimeric Mab were simultaneously added with U937 human monocytic cells (2×10^5) to the upper chamber. RPMI 1640 media containing 50 ng/ml human stromal cell-derived factor-1a (SDF-1a; R&D Systems) was placed in the lower chamber. After 12 h, cells that migrated to the lower chamber were collected and counted under a light microscope.

4. Cell enzyme-linked immunosorbent assay (ELISA)

Cell ELISA was performed as previously described (21). To investigate the effect of VCAM-1-D6 huMab on VCAM-1 downregulation on the surface of VCAM-1 expressing cells, TNFα-treated HUVECs (2×10^4) plated on 96-well plates were incubated with 20 μg/ml control IgG or VCAM-1-D6 huMab for the indicated times at 37°C. Cells were washed twice with ice-cold PBS, blocked in 3% (w/v) BSA in PBS for 1 h at 4°C, and incubated with mouse anti-VCAM-1 antibody (1:1000; Abcam) for 2 h at 4°C. Then, cells were incubated with HRP-conjugated anti-mouse IgG (1:5000; Santa Cruz Biotechnology) for 1 h at 4°C. After several washes with PBS, 100 ml TMB substrate solution was added to each well. Optical density was measured at 450 nm using a microtiter plate reader.
